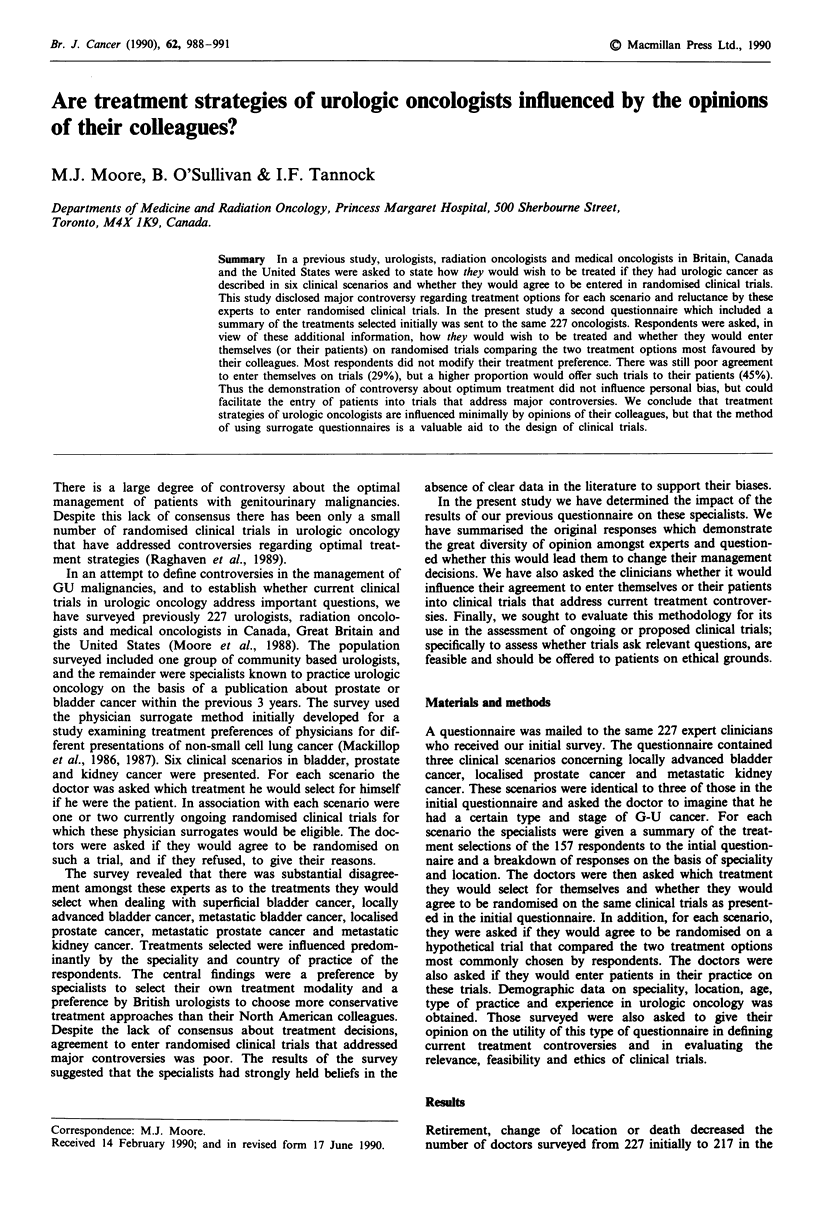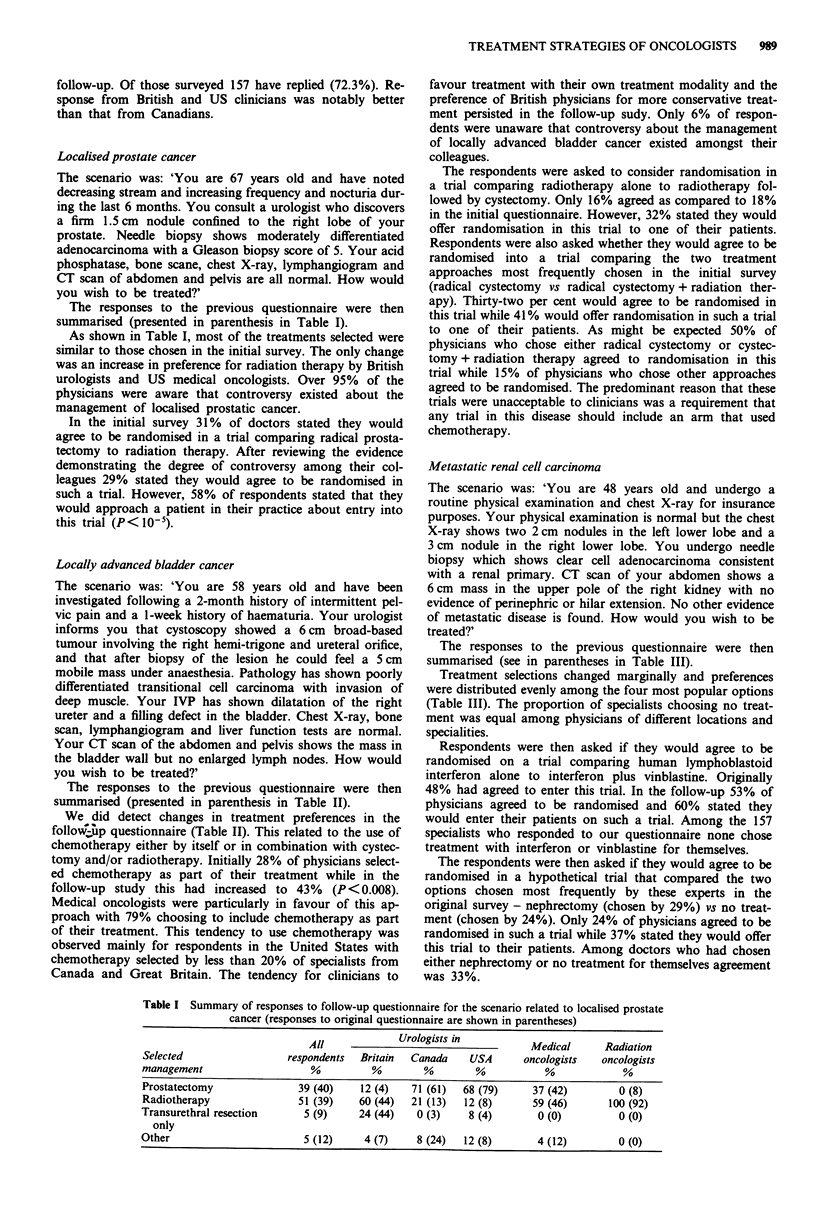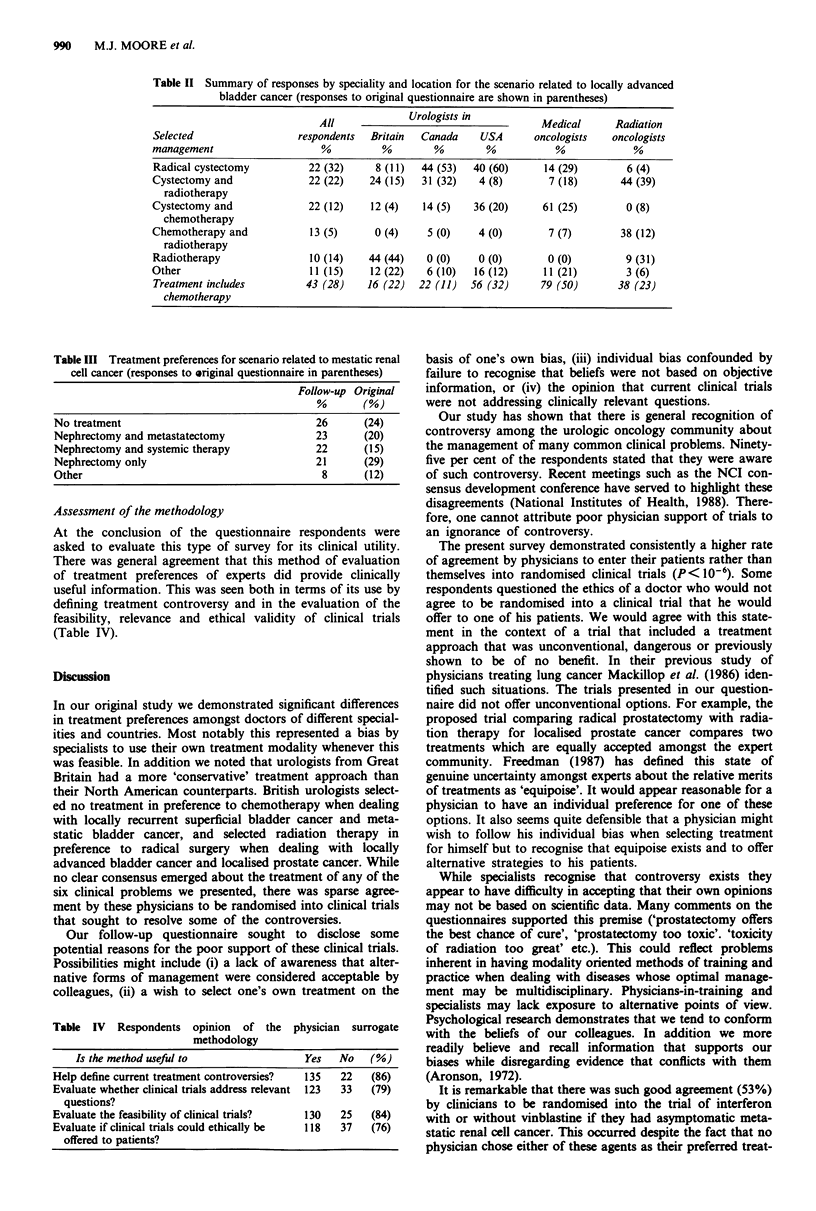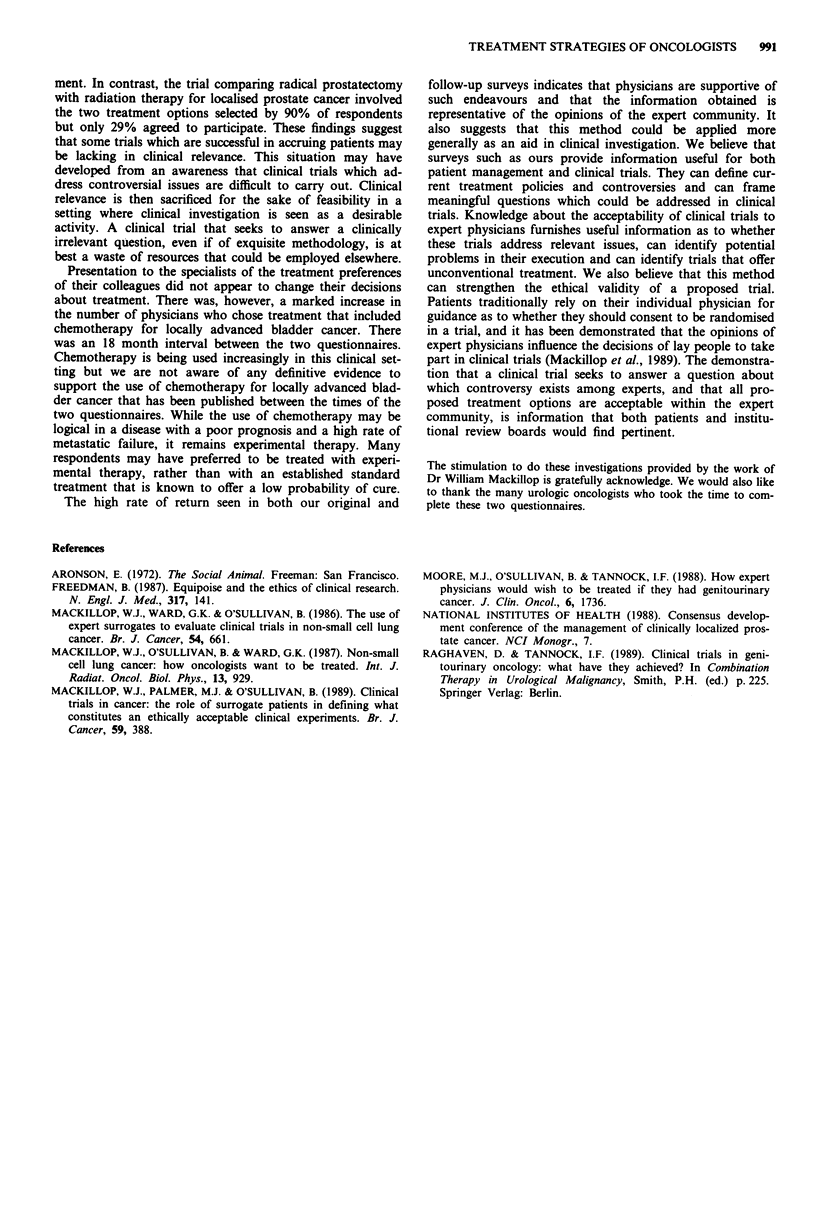# Are treatment strategies of urologic oncologists influenced by the opinions of their colleagues?

**DOI:** 10.1038/bjc.1990.422

**Published:** 1990-12

**Authors:** M. J. Moore, B. O'Sullivan, I. F. Tannock

**Affiliations:** Department of Medicine, Princess Margaret Hospital, Toronto, Canada.

## Abstract

In a previous study, urologists, radiation oncologists and medical oncologists in Britain, Canada and the United States were asked to state how they would wish to be treated if they had urologic cancer as described in six clinical scenarios and whether they would agree to be entered in randomised clinical trials. This study disclosed major controversy regarding treatment options for each scenario and reluctance by these experts to enter randomised clinical trials. In the present study a second questionnaire which included a summary of the treatments selected initially was sent to the same 227 oncologists. Respondents were asked, in view of these additional information, how they would wish to be treated and whether they would enter themselves (or their patients) on randomised trials comparing the two treatment options most favoured by their colleagues. Most respondents did not modify their treatment preference. There was still poor agreement to enter themselves on trials (29%), but a higher proportion would offer such trials to their patients (45%). Thus the demonstration of controversy about optimum treatment did not influence personal bias, but could facilitate the entry of patients into trials that address major controversies. We conclude that treatment strategies of urologic oncologists are influenced minimally by opinions of their colleagues, but that the method of using surrogate questionnaires is a valuable aid to the design of clinical trials.


Br. J. Cancer (1990), 62, 988-991 © Macmillan Press Ltd., 1990

### Are treatment strategies of urologic oncologists influenced by the opinions of their colleagues?

M.J. Moore, B. O'Sullivan & I.F. Tannock

*Departments of Medicine and Radiation Oncology, Princess Margaret Hospital, 500 Sherbourne Street, Toronto, M4X 1K9, Canada.*

**Summary** In a previous study, urologists, radiation oncologists and medical oncologists in Britain, Canada and the United States were asked to state how *they* would wish to be treated if they had urologic cancer as described in six clinical scenarios and whether they would agree to be entered in randomised clinical trials. This study disclosed major controversy regarding treatment options for each scenario and reluctance by these experts to enter randomised clinical trials. In the present study a second questionnaire which included a summary of the treatments selected initially was sent to the same 227 oncologists. Respondents were asked, in view of these additional information, how *they* would wish to be treated and whether they would enter themselves (or their patients) on randomised trials comparing the two treatment options most favoured by their colleagues. Most respondents did not modify their treatment preference. There was still poor agreement to enter themselves on trials (29%), but a higher proportion would offer such trials to their patients (45%). Thus the demonstration of controversy about optimum treatment did not influence personal bias, but could facilitate the entry of patients into trials that address major controversies. We conclude that treatment strategies of urologic oncologists are influenced minimally by opinions of their colleagues, but that the method of using surrogate questionnaires is a valuable aid to the design of clinical trials.

There is a large degree of controversy about the optimal management of patients with genitourinary malignancies. Despite this lack of consensus there has been only a small number of randomised clinical trials in urologic oncology that have addressed controversies regarding optimal treatment strategies (Raghaven *et al.*, 1989).

In an attempt to define controversies in the management of GU malignancies, and to establish whether current clinical trials in urologic oncology address important questions, we have surveyed previously 227 urologists, radiation oncologists and medical oncologists in Canada, Great Britain and the United States (Moore *et al.*, 1988). The population surveyed included one group of community based urologists, and the remainder were specialists known to practice urologic oncology on the basis of a publication about prostate or bladder cancer within the previous 3 years. The survey used the physician surrogate method initially developed for a study examining treatment preferences of physicians for different presentations of non-small cell lung cancer (Mackillop *et al.*, 1986, 1987). Six clinical scenarios in bladder, prostate and kidney cancer were presented. For each scenario the doctor was asked which treatment he would select for himself if he were the patient. In association with each scenario were one or two currently ongoing randomised clinical trials for which these physician surrogates would be eligible. The doctors were asked if they would agree to be randomised on such a trial, and if they refused, to give their reasons.

The survey revealed that there was substantial disagreement amongst these experts as to the treatments they would select when dealing with superficial bladder cancer, locally advanced bladder cancer, metastatic bladder cancer, localised prostate cancer, metastatic prostate cancer and metastatic kidney cancer. Treatments selected were influenced predominantly by the speciality and country of practice of the respondents. The central findings were a preference by specialists to select their own treatment modality and a preference by British urologists to choose more conservative treatment approaches than their North American colleagues. Despite the lack of consensus about treatment decisions, agreement to enter randomised clinical trials that addressed major controversies was poor. The results of the survey suggested that the specialists had strongly held beliefs in the absence of clear data in the literature to support their biases.

In the present study we have determined the impact of the results of our previous questionnaire on these specialists. We have summarised the original responses which demonstrate the great diversity of opinion amongst experts and questioned whether this would lead them to change their management decisions. We have also asked the clinicians whether it would influence their agreement to enter themselves or their patients into clinical trials that address current treatment controversies. Finally, we sought to evaluate this methodology for its use in the assessment of ongoing or proposed clinical trials; specifically to assess whether trials ask relevant questions, are feasible and should be offered to patients on ethical grounds.

### Materials and methods

A questionnaire was mailed to the same 227 expert clinicians who received our initial survey. The questionnaire contained three clinical scenarios concerning locally advanced bladder cancer, localised prostate cancer and metastatic kidney cancer. These scenarios were identical to three of those in the initial questionnaire and asked the doctor to imagine that he had a certain type and stage of G-U cancer. For each scenario the specialists were given a summary of the treatment selections of the 157 respondents to the intial questionnaire and a breakdown of responses on the basis of speciality and location. The doctors were then asked which treatment they would select for themselves and whether they would agree to be randomised on the same clinical trials as presented in the initial questionnaire. In addition, for each scenario, they were asked if they would agree to be randomised on a hypothetical trial that compared the two treatment options most commonly chosen by respondents. The doctors were also asked if they would enter patients in their practice on these trials. Demographic data on speciality, location, age, type of practice and experience in urologic oncology was obtained. Those surveyed were also asked to give their opinion on the utility of this type of questionnaire in defining current treatment controversies and in evaluating the relevance, feasibility and ethics of clinical trials.

### Results

Retirement, change of location or death decreased the number of doctors surveyed from 227 initially to 217 in the

Correspondence: M.J. Moore.
Received 14 February 1990; and in revised form 17 June 1990.

follow-up. Of those surveyed 157 have replied (72.3%). Response from British and US clinicians was notably better than that from Canadians.

*Localised prostate cancer*

The scenario was: 'You are 67 years old and have noted decreasing stream and increasing frequency and nocturia during the last 6 months. You consult a urologist who discovers a firm 1.5 cm nodule confined to the right lobe of your prostate. Needle biopsy shows moderately differentiated adenocarcinoma with a Gleason biopsy score of 5. Your acid phosphatase, bone scane, chest X-ray, lymphangiogram and CT scan of abdomen and pelvis are all normal. How would you wish to be treated?'

The responses to the previous questionnaire were then summarised (presented in parenthesis in Table I).

As shown in Table I, most of the treatments selected were similar to those chosen in the initial survey. The only change was an increase in preference for radiation therapy by British urologists and US medical oncologists. Over 95% of the physicians were aware that controversy existed about the management of localised prostatic cancer.

In the initial survey 31% of doctors stated they would agree to be randomised in a trial comparing radical prostatectomy to radiation therapy. After reviewing the evidence demonstrating the degree of controversy among their colleagues 29% stated they would agree to be randomised in such a trial. However, 58% of respondents stated that they would approach a patient in their practice about entry into this trial ($P < 10^{-5}$).

*Locally advanced bladder cancer*

The scenario was: 'You are 58 years old and have been investigated following a 2-month history of intermittent pelvic pain and a 1-week history of haematuria. Your urologist informs you that cystoscopy showed a 6 cm broad-based tumour involving the right hemi-trigone and ureteral orifice, and that after biopsy of the lesion he could feel a 5 cm mobile mass under anaesthesia. Pathology has shown poorly differentiated transitional cell carcinoma with invasion of deep muscle. Your IVP has shown dilatation of the right ureter and a filling defect in the bladder. Chest X-ray, bone scan, lymphangiogram and liver function tests are normal. Your CT scan of the abdomen and pelvis shows the mass in the bladder wall but no enlarged lymph nodes. How would you wish to be treated?'

The responses to the previous questionnaire were then summarised (presented in parenthesis in Table II).

We did detect changes in treatment preferences in the follow-up questionnaire (Table II). This related to the use of chemotherapy either by itself or in combination with cystectomy and/or radiotherapy. Initially 28% of physicians selected chemotherapy as part of their treatment while in the follow-up study this had increased to 43% ($P < 0.008$). Medical oncologists were particularly in favour of this approach with 79% choosing to include chemotherapy as part of their treatment. This tendency to use chemotherapy was observed mainly for respondents in the United States with chemotherapy selected by less than 20% of specialists from Canada and Great Britain. The tendency for clinicians to favour treatment with their own treatment modality and the preference of British physicians for more conservative treatment persisted in the follow-up sudy. Only 6% of respondents were unaware that controversy about the management of locally advanced bladder cancer existed amongst their colleagues.

The respondents were asked to consider randomisation in a trial comparing radiotherapy alone to radiotherapy followed by cystectomy. Only 16% agreed as compared to 18% in the initial questionnaire. However, 32% stated they would offer randomisation in this trial to one of their patients. Respondents were also asked whether they would agree to be randomised into a trial comparing the two treatment approaches most frequently chosen in the initial survey (radical cystectomy *vs* radical cystectomy + radiation therapy). Thirty-two per cent would agree to be randomised in this trial while 41% would offer randomisation in such a trial to one of their patients. As might be expected 50% of physicians who chose either radical cystectomy or cystectomy + radiation therapy agreed to randomisation in this trial while 15% of physicians who chose other approaches agreed to be randomised. The predominant reason that these trials were unacceptable to clinicians was a requirement that any trial in this disease should include an arm that used chemotherapy.

*Metastatic renal cell carcinoma*

The scenario was: 'You are 48 years old and undergo a routine physical examination and chest X-ray for insurance purposes. Your physical examination is normal but the chest X-ray shows two 2 cm nodules in the left lower lobe and a 3 cm nodule in the right lower lobe. You undergo needle biopsy which shows clear cell adenocarcinoma consistent with a renal primary. CT scan of your abdomen shows a 6 cm mass in the upper pole of the right kidney with no evidence of perinephric or hilar extension. No other evidence of metastatic disease is found. How would you wish to be treated?'

The responses to the previous questionnaire were then summarised (see in parentheses in Table III).

Treatment selections changed marginally and preferences were distributed evenly among the four most popular options (Table III). The proportion of specialists choosing no treatment was equal among physicians of different locations and specialities.

Respondents were then asked if they would agree to be randomised on a trial comparing human lymphoblastoid interferon alone to interferon plus vinblastine. Originally 48% had agreed to enter this trial. In the follow-up 53% of physicians agreed to be randomised and 60% stated they would enter their patients on such a trial. Among the 157 specialists who responded to our questionnaire none chose treatment with interferon or vinblastine for themselves.

The respondents were then asked if they would agree to be randomised in a hypothetical trial that compared the two options chosen most frequently by these experts in the original survey – nephrectomy (chosen by 29%) *vs* no treatment (chosen by 24%). Only 24% of physicians agreed to be randomised in such a trial while 37% stated they would offer this trial to their patients. Among doctors who had chosen either nephrectomy or no treatment for themselves agreement was 33%.

**Table I** Summary of responses to follow-up questionnaire for the scenario related to localised prostate cancer (responses to original questionnaire are shown in parentheses)

| Selected management | All respondents % | Urologists in Britain % | Urologists in Canada % | Urologists in USA % | Medical oncologists % | Radiation oncologists % |
|---|---|---|---|---|---|---|
| Prostatectomy | 39 (40) | 12 (4) | 71 (61) | 68 (79) | 37 (42) | 0 (8) |
| Radiotherapy | 51 (39) | 60 (44) | 21 (13) | 12 (8) | 59 (46) | 100 (92) |
| Transurethral resection only | 5 (9) | 24 (44) | 0 (3) | 8 (4) | 0 (0) | 0 (0) |
| Other | 5 (12) | 4 (7) | 8 (24) | 12 (8) | 4 (12) | 0 (0) |

Table II Summary of responses by speciality and location for the scenario related to locally advanced bladder cancer (responses to original questionnaire are shown in parentheses)

| Selected management | All respondents % | Urologists in Britain % | Urologists in Canada % | Urologists in USA % | Medical oncologists % | Radiation oncologists % |
|---|---|---|---|---|---|---|
| Radical cystectomy | 22 (32) | 8 (11) | 44 (53) | 40 (60) | 14 (29) | 6 (4) |
| Cystectomy and radiotherapy | 22 (22) | 24 (15) | 31 (32) | 4 (8) | 7 (18) | 44 (39) |
| Cystectomy and chemotherapy | 22 (12) | 12 (4) | 14 (5) | 36 (20) | 61 (25) | 0 (8) |
| Chemotherapy and radiotherapy | 13 (5) | 0 (4) | 5 (0) | 4 (0) | 7 (7) | 38 (12) |
| Radiotherapy | 10 (14) | 44 (44) | 0 (0) | 0 (0) | 0 (0) | 9 (31) |
| Other | 11 (15) | 12 (22) | 6 (10) | 16 (12) | 11 (21) | 3 (6) |
| *Treatment includes chemotherapy* | *43 (28)* | *16 (22)* | *22 (11)* | *56 (32)* | *79 (50)* | *38 (23)* |

Table III Treatment preferences for scenario related to mestatic renal cell cancer (responses to original questionnaire in parentheses)

| | Follow-up % | Original (%) |
|---|---|---|
| No treatment | 26 | (24) |
| Nephrectomy and metastatectomy | 23 | (20) |
| Nephrectomy and systemic therapy | 22 | (15) |
| Nephrectomy only | 21 | (29) |
| Other | 8 | (12) |

*Assessment of the methodology*

At the conclusion of the questionnaire respondents were asked to evaluate this type of survey for its clinical utility. There was general agreement that this method of evaluation of treatment preferences of experts did provide clinically useful information. This was seen both in terms of its use by defining treatment controversy and in the evaluation of the feasibility, relevance and ethical validity of clinical trials (Table IV).

### Discussion

In our original study we demonstrated significant differences in treatment preferences amongst doctors of different specialities and countries. Most notably this represented a bias by specialists to use their own treatment modality whenever this was feasible. In addition we noted that urologists from Great Britain had a more 'conservative' treatment approach than their North American counterparts. British urologists selected no treatment in preference to chemotherapy when dealing with locally recurrent superficial bladder cancer and metastatic bladder cancer, and selected radiation therapy in preference to radical surgery when dealing with locally advanced bladder cancer and localised prostate cancer. While no clear consensus emerged about the treatment of any of the six clinical problems we presented, there was sparse agreement by these physicians to be randomised into clinical trials that sought to resolve some of the controversies.

Our follow-up questionnaire sought to disclose some potential reasons for the poor support of these clinical trials. Possibilities might include (i) a lack of awareness that alternative forms of management were considered acceptable by colleagues, (ii) a wish to select one's own treatment on the basis of one's own bias, (iii) individual bias confounded by failure to recognise that beliefs were not based on objective information, or (iv) the opinion that current clinical trials were not addressing clinically relevant questions.

Table IV Respondents opinion of the physician surrogate methodology

| *Is the method useful to* | *Yes* | *No* | *(%)* |
|---|---|---|---|
| Help define current treatment controversies? | 135 | 22 | (86) |
| Evaluate whether clinical trials address relevant questions? | 123 | 33 | (79) |
| Evaluate the feasibility of clinical trials? | 130 | 25 | (84) |
| Evaluate if clinical trials could ethically be offered to patients? | 118 | 37 | (76) |

Our study has shown that there is general recognition of controversy among the urologic oncology community about the management of many common clinical problems. Ninety-five per cent of the respondents stated that they were aware of such controversy. Recent meetings such as the NCI consensus development conference have served to highlight these disagreements (National Institutes of Health, 1988). Therefore, one cannot attribute poor physician support of trials to an ignorance of controversy.

The present survey demonstrated consistently a higher rate of agreement by physicians to enter their patients rather than themselves into randomised clinical trials ($P < 10^{-6}$). Some respondents questioned the ethics of a doctor who would not agree to be randomised into a clinical trial that he would offer to one of his patients. We would agree with this statement in the context of a trial that included a treatment approach that was unconventional, dangerous or previously shown to be of no benefit. In their previous study of physicians treating lung cancer Mackillop *et al.* (1986) identified such situations. The trials presented in our questionnaire did not offer unconventional options. For example, the proposed trial comparing radical prostatectomy with radiation therapy for localised prostate cancer compares two treatments which are equally accepted amongst the expert community. Freedman (1987) has defined this state of genuine uncertainty amongst experts about the relative merits of treatments as 'equipoise'. It would appear reasonable for a physician to have an individual preference for one of these options. It also seems quite defensible that a physician might wish to follow his individual bias when selecting treatment for himself but to recognise that equipoise exists and to offer alternative strategies to his patients.

While specialists recognise that controversy exists they appear to have difficulty in accepting that their own opinions may not be based on scientific data. Many comments on the questionnaires supported this premise ('prostatectomy offers the best chance of cure', 'prostatectomy too toxic'. 'toxicity of radiation too great' etc.). This could reflect problems inherent in having modality oriented methods of training and practice when dealing with diseases whose optimal management may be multidisciplinary. Physicians-in-training and specialists may lack exposure to alternative points of view. Psychological research demonstrates that we tend to conform with the beliefs of our colleagues. In addition we more readily believe and recall information that supports our biases while disregarding evidence that conflicts with them (Aronson, 1972).

It is remarkable that there was such good agreement (53%) by clinicians to be randomised into the trial of interferon with or without vinblastine if they had asymptomatic metastatic renal cell cancer. This occurred despite the fact that no physician chose either of these agents as their preferred treatment. In contrast, the trial comparing radical prostatectomy with radiation therapy for localised prostate cancer involved the two treatment options selected by 90% of respondents but only 29% agreed to participate. These findings suggest that some trials which are successful in accruing patients may be lacking in clinical relevance. This situation may have developed from an awareness that clinical trials which address controversial issues are difficult to carry out. Clinical relevance is then sacrificed for the sake of feasibility in a setting where clinical investigation is seen as a desirable activity. A clinical trial that seeks to answer a clinically irrelevant question, even if of exquisite methodology, is at best a waste of resources that could be employed elsewhere.

Presentation to the specialists of the treatment preferences of their colleagues did not appear to change their decisions about treatment. There was, however, a marked increase in the number of physicians who chose treatment that included chemotherapy for locally advanced bladder cancer. There was an 18 month interval between the two questionnaires. Chemotherapy is being used increasingly in this clinical setting but we are not aware of any definitive evidence to support the use of chemotherapy for locally advanced bladder cancer that has been published between the times of the two questionnaires. While the use of chemotherapy may be logical in a disease with a poor prognosis and a high rate of metastatic failure, it remains experimental therapy. Many respondents may have preferred to be treated with experimental therapy, rather than with an established standard treatment that is known to offer a low probability of cure.

The high rate of return seen in both our original and follow-up surveys indicates that physicians are supportive of such endeavours and that the information obtained is representative of the opinions of the expert community. It also suggests that this method could be applied more generally as an aid in clinical investigation. We believe that surveys such as ours provide information useful for both patient management and clinical trials. They can define current treatment policies and controversies and can frame meaningful questions which could be addressed in clinical trials. Knowledge about the acceptability of clinical trials to expert physicians furnishes useful information as to whether these trials address relevant issues, can identify potential problems in their execution and can identify trials that offer unconventional treatment. We also believe that this method can strengthen the ethical validity of a proposed trial. Patients traditionally rely on their individual physician for guidance as to whether they should consent to be randomised in a trial, and it has been demonstrated that the opinions of expert physicians influence the decisions of lay people to take part in clinical trials (Mackillop *et al.*, 1989). The demonstration that a clinical trial seeks to answer a question about which controversy exists among experts, and that all proposed treatment options are acceptable within the expert community, is information that both patients and institutional review boards would find pertinent.

The stimulation to do these investigations provided by the work of Dr William Mackillop is gratefully acknowledge. We would also like to thank the many urologic oncologists who took the time to complete these two questionnaires.

### References

ARONSON, E. (1972). *The Social Animal*. Freeman: San Francisco.

FREEDMAN, B. (1987). Equipoise and the ethics of clinical research. *N. Engl. J. Med.*, **317**, 141.

MACKILLOP, W.J., WARD, G.K. & O'SULLIVAN, B. (1986). The use of expert surrogates to evaluate clinical trials in non-small cell lung cancer. *Br. J. Cancer*, **54**, 661.

MACKILLOP, W.J., O'SULLIVAN, B. & WARD, G.K. (1987). Non-small cell lung cancer: how oncologists want to be treated. *Int. J. Radiat. Oncol. Biol. Phys.*, **13**, 929.

MACKILLOP, W.J., PALMER, M.J. & O'SULLIVAN, B. (1989). Clinical trials in cancer: the role of surrogate patients in defining what constitutes an ethically acceptable clinical experiments. *Br. J. Cancer*, **59**, 388.

MOORE, M.J., O'SULLIVAN, B. & TANNOCK, I.F. (1988). How expert physicians would wish to be treated if they had genitourinary cancer. *J. Clin. Oncol.*, **6**, 1736.

NATIONAL INSTITUTES OF HEALTH (1988). Consensus development conference of the management of clinically localized prostate cancer. *NCI Monogr.*, 7.

RAGHAVEN, D. & TANNOCK, I.F. (1989). Clinical trials in genitourinary oncology: what have they achieved? In *Combination Therapy in Urological Malignancy*, Smith, P.H. (ed.) p. 225. Springer Verlag: Berlin.